# Identification of whole‐genome significant single nucleotide polymorphisms in candidate genes associated with body conformation traits in Chinese Holstein cattle

**DOI:** 10.1111/age.12865

**Published:** 2019-10-21

**Authors:** Zhengui Yan, Zhonghua Wang, Qin Zhang, Shujian Yue, Bin Yin, Yunliang Jiang, Kerong Shi

**Affiliations:** ^1^ College of Animal Science and Technology Shandong Key Laboratory of Animal Bioengineering and Disease Prevention Shandong Agricultural University Taian Shandong 271018 China

GWAS has been proven to be a powerful tool for detecting genetic variants associated with economically important traits such as production,^1^, ^2^ body conformation^3^, ^4^ and reproduction traits,^5^ and diseases.^6^ The objective of this study was to identify SNPs with significant association effects on body conformation traits in Chinese Holstein through the GWAS approach.

The experimental population consisted of 445 Chinese Holstein cows, with parity no. 2, raised at four farms in Shandong province of China. Hair follicle samples were collected individually within four days in the same season. In total, 29 body conformation traits were analyzed by GWAS. There were 23 phenotypes measured individually on 1–9 scores, which were body height, height at front end, body depth, chest width, stature, angularity, rump angle (RA), pin width, loin strength, foot angle, heel depth, bone quality, rear legs – side view, rear legs – rear view, udder depth, udder texture, median suspensory, fore udder attachment, front teat placement, attachment height, attachment width (AW), rear teat placement and teat length. The remaining six functional body conformation traits, measured on 1–100 scores, were stature score, rump system score, feet and legs score, udder system score, mammary system score and conformation final score (FS). The definitions of these conformation traits for cattle are available on the website of Canadian Dairy Network (https://www.cdn.ca/articles.php). The summary statistics of these phenotypes are listed in Table S1.

Each experimental animal was genotyped by the GGP BovineLD V3 SNP chip, containing 26 151 public SNPs. Because all of the animals in these study populations were female, the SNPs from the X chromosome were also counted. The data quality control procedure excluded individuals with more than 10% missing genotypes and SNPs with a call rate of less than 90%, MAF less than 3% or *P*‐value of the Hardy–Weinberg Equilibrium test less than 1.0E − 6. Finally, 421 animals with 20 632 SNP genotypes were retained for the subsequent GWAS analysis. Physical map length, the number of SNPs and SNP density on each chromosome based on reference genome UMD3.1, before and after the data cleaning procedure, are shown in Table S2. Results of pair‐wise LD analysis of the 421 animals from four different farms showed high similarity of LD patterns among the whole population, indicating that similar breeding histories were shared by the four subpopulations (data not provided).

A fixed‐effects linear regression model was used to carry out the GWAS according to a previous method.^2^, ^7^ Bonferroni correction for the genome‐wide significance and suggestive thresholds were computed to be 2.42E − 06 (= 0.05/20 632) and 4.85E − 05 (= 1/20 632), respectively.

The GWAS based on the mixed‐effect linear regression model identified 79 SNPs with genome‐wide significant (*P *<* *0.05) association effects on 14 body conformation traits (Table 1, Table S3). Of these SNPs, 28 SNPs were found to be associated with RA, and 14 SNPs associated with FS (Table 1, Fig. S1). We also found six single SNPs that were associated with multiple body conformation traits of dairy cow. These SNPs were BovineHD0600000461, BovineHD1900007686, ARS‐BFGL‐NGS‐41612, BovineHD1700010514, BovineHD3000037672 and ARS‐BFGL‐NGS‐109467 (their detailed information is provided in Table S4). In this study, the DNA region from 44.6 to 65.4 Mb of chromosome 18 was identified to be associated with four different body conformation traits (AW, FS, mammary system score and RA) of dairy cows (Table 2, Table S3). This coincided with previously reported QTL or DNA regions associated with calving performance and udder traits of dairy cattle.^8^, ^9^, ^10^ Among these SNPs, Hapmap57004‐rs29011610, located near *MON1B* (MON1 secretory trafficking family member B) gene, were also found to be associated with length of productive life in Holstein cows.^11^ ARS‐BFGL‐NGS‐116541, which was located within the *LIG1* (Ligase I) gene region, was reported to be associated with body weight at birth.^12^ Similarly, DNA regions on chromosomes 21, 25 and X were also identified to be significantly associated with multiple body conformation traits (Table 2, Table S3). In particular, six SNPs in the small region from 57.55 to 57.62 Mb on the chromosome 21 were found to be associated with AW and FS in the study, and these SNPs were all located within or near the gene region of *SLC24A4* (Table S5). Interestingly, *SLC24A5* and *SLC19A2*, members of solute carrier family proteins with *SLC24A4*, were also found to be associated with RA and FS, respectively (Table 1). Additionally, UA‐IFASA‐6670, located within the region of *GABARAPL1* [GABA (A) receptor‐associated protein like 1] gene, were found to be significantly associated with median suspensory in this study. It was reported previously to affect udder attachment and height.^13^, ^14^ Also, BovineHD2200013812 was identified to have genetic effects on RA. This SNP was located within the *CACNA1D* (calcium channel alpha 1D subunit) gene region. Coincidentally, this SNP was also reported to be associated with the length of productive life and udder cleft.^14^

**Table 1:** The significant SNPs and genes that are associated genome‐wide with body conformation traits

Category	Trait	Chromosome^1^	Position	SNP name	*P*‐Value	Nearest gene	Distance^2^	Reference	Alteration	MAF
Dairy strength	Angularity	30	110222328	BTA‐116883‐no‐rs	1.56E − 06	*LOC786124*	D:27320	G	A	0.2404
		30	116273044	BovineHD3000032546	6.55E − 07	*LOC537655*	Within	C	A	0.3061
		30	132705219	BovineHD3000037672	5.10E − 08	*LOC786725*	U:54284	T	C	0.449
	Stature	10	90906002	Hapmap39512‐BTA‐79353	5.11E − 07			T	G	0.2744
		19	26049717	BovineHD1900007686	2.15E − 06	*AIPL1*	D:7711	T	C	0.03628
	Chest width	17	38504014	BovineHD1700010514	1.34E − 07	*LOC512119*	D:176780	T	C	0.09545
	Body height	11	80730546	ARS‐BFGL‐NGS‐41612	4.93E − 07	*KCNS3*	U:112015	G	A	0.1485
		11	105064528	BovineHD1100030541	1.50E − 06	*LOC789076*	U:83296	C	T	0.4728
		13	21301875	BovineHD1300006183	4.26E − 07	*LOC524240*	Within	G	A	0.3515
		23	39248351	BovineHD2300011340	2.39E − 06	*NHLRC1*	D:7508	T	C	0.4341
		27	39335460	Hapmap38550‐BTA‐98603	1.66E − 06	*LRRC3B*	D:101345	C	A	0.3497
	Stature score	10	45053776	BovineHD1000013564	9.37E − 07	*NID2*	D:67117	G	T	0.2324
		17	38504014	BovineHD1700010514	2.42E − 08	*LOC512119*	D:176780	T	C	0.09545
Mammary system	Attachment width	9	20356212	BTA‐85319‐no‐rs	7.16E − 07	*BCKDHB*	D:21051	T	C	0.2614
		18	65402237	BovineHD1800019049	4.75E − 07	*LOC789374*	Within	C	T	0.2143
		19	24263948	BovineHD1900006998	1.35E − 06	*LOC786649*	U:52976	C	T	0.4932
		21	57583470	BovineHD2100016545	2.26E − 06	*SLC24A4*	U:12991	A	G	0.4478
		21	57584406	BovineHD2100016546	2.33E − 06	*SLC24A4*	U:12055	A	C	0.4477
		21	57587712	BovineHD2100016549	2.26E − 06	*SLC24A4*	U:8749	C	T	0.4478
		21	57620878	BovineHD2100016561	9.13E − 07	*SLC24A4*	Within	G	A	0.2823
		21	57623572	BovineHD2100016563	9.13E − 07	*SLC24A4*	Within	G	A	0.2823
		26	37203584	ARS‐BFGL‐NGS‐8275	8.12E − 07	*LOC531271*	Within	A	G	0.2381
	Median suspensory	5	100206147	UA‐IFASA‐6670	6.37E − 07	*GABARAPL1*	Within	C	T	0.398
		9	93370986	BovineHD0900026424	5.03E − 07	*NOX3*	U:2759	T	C	0.325
		17	73901259	BovineHD1700021616	9.77E − 07	*LOC531152*	D:1528	T	C	0.1462
		30	138304543	BovineHD3000039710	5.31E − 07	*LOC782196*	U:18403	T	C	0.213
	Mammary system score	13	28331553	ARS‐BFGL‐NGS‐109467	5.49E − 07	*SEPHS1*	D:16954	A	G	0.4388
		15	43538866	ARS‐BFGL‐NGS‐115625	2.03E − 06	*SWAP70*	Within	G	A	0.4467
		18	55956772	ARS‐BFGL‐NGS‐60829	6.86E − 07	*NUCB1*	D:1160	C	T	0.4172
		30	132705219	BovineHD3000037672	7.03E − 08	*LOC786725*	U:54284	T	C	0.449
	Rear teat placement	9	25459892	BTA‐83107‐no‐rs	1.10E − 06	*MIR2284O*	U:14492	A	G	0.3307
	Udder depth	25	35623801	ARS‐BFGL‐NGS‐28167	2.16E − 06	*CUX1*	D:12155	A	G	0.4023
		25	36266951	BovineHD2500010029	1.27E − 07	*LOC100298352*	Within	T	C	0.356
	Udder system score	6	1770665	BovineHD0600000461	1.03E − 07	*1‐Mar*	Within	G	A	0.39
Rump structure	Loin strength	5	61620118	BovineHD0500017277	2.90E − 07	*NEDD1*	D:121565	T	G	0.4853
		7	53932886	ARS‐BFGL‐NGS‐20197	5.71E − 07	*PCDHB6*	U:892	T	C	0.4966
		28	46248750	BovineHD2800013502	4.71E − 07	*LOC100141022*	D:32551	T	C	0.2523
	Rump angle	1	5519845	BTB‐00003652	1.76E − 06	*GRIK1*	Within	C	T	0.25
		1	68909418	BovineHD0100019488	4.88E − 07	*CCDC14*	Within	A	G	0.4182
		1	123851563	BovineHD0100034972	1.97E − 06			T	C	0.2011
		1	142967164	BovineHD0100041062	2.03E − 06	*BACE2*	Within	G	A	0.4354
		2	127566752	BovineHD0200037025	6.11E − 07	*PDIK1L*	Within	G	A	0.3417
		4	10148342	Hapmap35652‐SCAFFOLD151622_1051	4.57E − 07	*LOC100295705*	D:88944	C	T	0.2268
		6	83512619	BTA‐107087‐no‐rs	2.18E − 06	*LOC100298985*	U:19994	G	A	0.3243
		6	87715723	Hapmap38371‐BTA‐105598	1.58E − 06	*AMBN*	D:9991	C	A	0.3356
		7	83260664	BovineHD0700024393	4.32E − 09	*MSH3*	D:33418	G	A	0.1746
		7	83757564	BovineHD0700024587	1.14E − 07	*SSBP2*	Within	C	A	0.11
		7	91507089	ARS‐BFGL‐NGS‐118534	2.83E − 07			G	A	0.2761
		8	101664818	BovineHD0800030195	2.25E − 06	*SVEP1*	Within	G	A	0.1236
		9	81329823	BTA‐106078‐no‐rs	9.84E − 07	*HIVEP2*	D:62953	A	G	0.4592
		10	43438784	BovineHD1000013067	8.09E − 08	*MAP4K5*	Within	G	A	0.3246
		10	62563388	BovineHD1000018043	7.73E − 07	*SLC24A5*	D:73734	T	C	0.4376
		10	73979984	Hapmap49737‐BTA‐75278	6.00E − 07	*PRKCH*	D:52860	A	G	0.04762
		18	55309510	ARS‐BFGL‐NGS‐116541	2.37E − 06	*LIG1*	Within	C	A	0.4487
		18	55514759	BovineHD1800016250	7.28E − 08	*SYNGR4*	Within	T	C	0.2494
		18	55621823	ARS‐BFGL‐NGS‐31529	2.12E − 06	*LMTK3*	Within	T	G	0.2426
		22	47989704	BovineHD2200013812	1.72E − 06	*CACNA1D*	Within	T	C	0.2727
		22	48408579	BovineHD2200013926	5.34E − 07	*RFT1*	Within	G	A	0.0839
		24	29554807	BovineHD2400008037	2.70E − 07	*LOC782418*	D:63567	G	A	0.2323
		25	31147780	Hapmap24744‐BTC‐028427	1.82E − 08	*LOC100301342*	Within	T	C	0.3898
		25	42364359	ARS‐BFGL‐NGS‐101981	1.32E − 06	*ADAP1*	D:1441	T	C	0.2823
		26	16504170	BovineHD2600004135	1.32E − 06	*LOC522146*	Within	A	G	0.1927
		29	7216410	BovineHD2900002021	5.13E − 07	*LOC100336861*	Within	G	A	0.1961
		30	2037499	BovineHD3000000680	2.28E − 06	*KLHL13*	Within	C	A	0.03855
		30	141936249	BTA‐21001‐no‐rs	2.16E − 06	*MSL3*	U:105193	C	T	0.1939
Final conformation score	Final score	6	1770665	BovineHD0600000461	1.75E − 06	*1‐Mar*	Within	G	A	0.39
		11	80730546	ARS‐BFGL‐NGS‐41612	4.61E − 07	*KCNS3*	U:112015	G	A	0.1485
		13	28331553	ARS‐BFGL‐NGS‐109467	1.80E − 06	*SEPHS1*	D:16954	A	G	0.4388
		16	37787772	ARS‐BFGL‐NGS‐34764	2.01E − 06	*NME7*	Within	G	A	0.4887
		16	37904090	BTB‐00637941	1.17E − 06	*SLC19A2*	Within	C	T	0.4863
		16	41384258	BovineHD1600011634	2.18E − 06	*LOC614226*	U:123143	T	C	0.4558
		18	4463083	Hapmap57004‐rs29011610	1.17E − 06	*MON1B*	D:5928	G	A	0.3129
		18	65405023	BovineHD1800019051	2.04E − 06	*LOC789374*	Within	A	G	0.1179
		19	26049717	BovineHD1900007686	7.31E − 07	*AIPL1*	D:7711	T	C	0.03628
		21	57552028	BovineHD2100016535	8.46E − 07	*SLC24A4*	U:44433	A	G	0.234
		25	39558290	BovineHD2500011031	2.24E − 06	*WIPI2*	U:7420	T	G	0.09524
		25	40192570	BovineHD4100017518	1.07E − 06	*SDK1*	Within	T	C	0.03061
		26	44324248	BovineHD2600012439	1.82E − 06	*OAT*	U:53908	C	A	0.3379
		30	12103258	BovineHD3000003945	8.38E − 07	*ACTRT1*	U:55118	G	A	0.1497

^1^ Chromosome 30 refers to X chromosome.

^2^ The distance from the SNP locus to the gene (unit: bp); D and U indicate that the SNP site is located downstream and upstream of the gene, respectively; ‘Within’ indicates that the SNP locus is located within the gene.

**Table 2:** DNA regions of chromosomes 18, 21, 25 and X containing SNPs that were significantly associated with body conformation traits of dairy cows

Chromosome	Trait	Position	SNP name	*P*‐Value	Nearest gene	Distance^1^	Reference	Alteration	MAF
18	AW	65402237	BovineHD1800019049	4.75E − 07	*LOC789374*	Within	C	T	0.2143
	FS	4463083	Hapmap57004‐rs29011610	1.17E − 06	*MON1B*	D: 5928	G	A	0.3129
	FS	65405023	BovineHD1800019051	2.04E − 06	*LOC789374*	Within	A	G	0.1179
	MSS	55956772	ARS‐BFGL‐NGS‐60829	6.86E − 07	*NUCB1*	D: 1160	C	T	0.4172
	RA	55309510	ARS‐BFGL‐NGS‐116541	2.37E − 06	*LIG1*	Within	C	A	0.4487
	RA	55514759	BovineHD1800016250	7.28E − 08	*SYNGR4*	Within	T	C	0.2494
	RA	55621823	ARS‐BFGL‐NGS‐31529	2.12E − 06	*LMTK3*	Within	T	G	0.2426
21	FS	57552028	BovineHD2100016535	8.46E − 07	*SLC24A4*	U: 44433	A	G	0.2340
	AW	57583470	BovineHD2100016545	2.26E − 06	*SLC24A4*	U: 12991	A	G	0.4478
	AW	57584406	BovineHD2100016546	2.33E − 06	*SLC24A4*	U: 12055	A	C	0.4477
	AW	57587712	BovineHD2100016549	2.26E − 06	*SLC24A4*	U: 8749	C	T	0.4478
	AW	57620878	BovineHD2100016561	9.13E − 07	*SLC24A4*	Within	G	A	0.2823
	AW	57623572	BovineHD2100016563	9.13E − 07	*SLC24A4*	Within	G	A	0.2823
25	RA	42364359	ARS‐BFGL‐NGS‐101981	1.32E − 06	*ADAP1*	D: 1441	T	C	0.2823
	UD	35623801	ARS‐BFGL‐NGS‐28167	2.16E − 06	*CUX1*	D: 12155	A	G	0.4023
	UD	36266951	BovineHD2500010029	1.27E − 07	*LOC100298352*	Within	T	C	0.3560
	RA	31147780	Hapmap24744‐BTC‐028427	1.82E − 08	*LOC100301342*	Within	T	C	0.3898
	FS	40192570	BovineHD4100017518	1.07E − 06	*SDK1*	Within	T	C	0.03061
X	FS	12103258	BovineHD3000003945	8.38E − 07	*ACTRT1*	U: 55118	G	A	0.1497
	RA	2037499	BovineHD3000000680	2.28E − 06	*KLHL13*	Within	C	A	0.03855
	AG	116273044	BovineHD3000032546	6.55E − 07	*LOC537655*	Within	C	A	0.3061
	MS	138304543	BovineHD3000039710	5.31E − 07	*LOC782196*	U: 18403	T	C	0.2130
	AG	110222328	BTA‐116883‐no‐rs	1.56E − 06	*LOC786124*	D: 27320	G	A	0.2404
	AG	132705219	BovineHD3000037672	5.10E − 08	*LOC786725*	U: 54284	T	C	0.4490
	MSS	132705219	BovineHD3000037672	7.03E − 08	*LOC786725*	U: 54284	T	C	0.4490
	RA	141936249	BTA‐21001‐no‐rs	2.16E − 06	*MSL3*	U: 105193	C	T	0.1939

AW, Attachment width; FS, conformation final score; MSS, mammary system score; RA, rump angle; UD, udder depth; AG, angularity; MS, median suspensory; MAF means minor allele frequency.

^1^ The distance from the SNP locus to the gene (unit: bp); D and U indicate that the SNP site is located downstream and upstream of the gene, respectively; ‘Within’ indicates that the SNP locus is located within the gene.

In summary, a GWAS using linear statistical model was conducted on 29 body conformation traits in a Chinese Holstein cattle population, and 79 SNPs were found to have genome‐wide‐significant (*P *<* *0.05) association effects on 14 body conformation traits. Among these significant SNPs, 74 of them are newly detected in this study, five have been reported in previous literature and 26 are located in genes and are worth further investigation to potentially identify the causative mutations underlying the QTL.

## Conflict of interest

The authors declare that there is no conflict of interest.

## Supporting information


**Figure S1** Genome‐wide plots of −log10 (*P*‐value) SNP association effects on body conformation traits of rump angle (RA, a) and final score (FS, b) obtained by mixed‐effect linear regression model.

**Table S1** Descriptive statistics of the 29 conformation traits used in the GWAS.
**Table S2** Distribution of SNP markers by chromosomes before and after quality control.
**Table S3** The chromosomal distribution of significant SNPs associated with body conformation traits.
**Table S4** The SNPs identified associated with multiple body conformation traits of dairy cows.
**Table S5** Multiple SNPs located in the *SLC24A4* gene region were significantly associated with body conformation traits of dairy cows.
