# Supplementary material for: Identification of whole‐genome significant single nucleotide polymorphisms in candidate genes associated with body conformation traits in Chinese Holstein cattle
Source: Anim Genet. 2019 Oct 21;51(1):141–6. doi: 10.1111/age.12865 (PMC7003999; doi:10.1111/age.12865)
Supplement: Supplementary file 1 — Figure S1 Genome‐wide plots of −log10 (P‐value) SNP association effects on body conformation traits of rump angle (RA, a) and final score (FS, b) obtained by mixed‐effect linear regression model. [file AGE-51-141-s001.pdf]

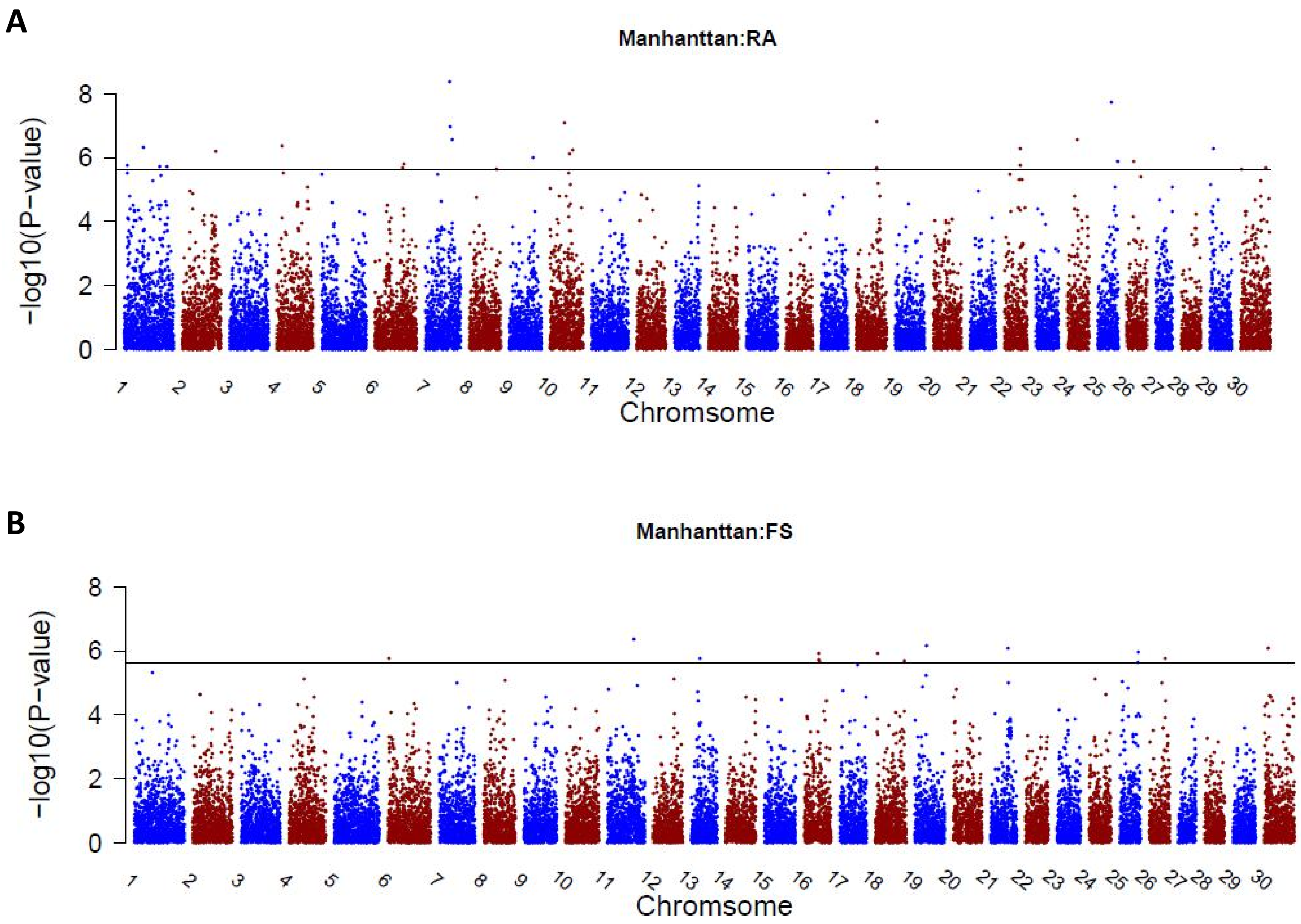

A
Manhanttan:RA
-log10(P-value)
0
2
4
6
8
1 2 3 4 5 6 7 8 9 10 11 12 13 14 15 16 17 18 19 20 21 22 23 24 25 26 27 28 29 30
Chromsome
B
Manhanttan:FS
-log10(P-value)
0
2
4
6
8
1 2 3 4 5 6 7 8 9 10 11 12 13 14 15 16 17 18 19 20 21 22 23 24 25 26 27 28 29 30
Chromsome

Figure S1. Genome-wide plots of –log10 (*P*-value) SNP association effects on body confirmation trait of rump angle (RA, A) and final sore (FS, B) obtained by mixed-effect linear regression model (MLM). The scale on the x-axis represents the physical length along each chromosome, wherein chromosome 30 refer to X chromosome, which was considered as an autosome because of overall female cattle in the studied population. The scale on the y-axis is the –log10 (*P*-value) SNP association effects. The solid line represents 5% of genome-wise significance with the corresponding raw *P*-value being equal to 2.42×10^{-6}.
