# Supplementary material for: Identification of whole‐genome significant single nucleotide polymorphisms in candidate genes associated with body conformation traits in Chinese Holstein cattle
Source: Anim Genet. 2019 Oct 21;51(1):141–6. doi: 10.1111/age.12865 (PMC7003999; doi:10.1111/age.12865)
Supplement: Supplementary file 2 — Table S1 Descriptive statistics of the 29 conformation traits used in the GWAS. Table S2 Distribution of SNP markers by chromosomes before and after quality control. Table S3 The chromosomal distribution of significant SNPs associated with body conformation traits. Table S4 The SNPs identified associated with multiple body conformation traits of dairy cows. Table S5 Multiple SNPs located in the SLC24A4 gene region were significantly associated with body conformation traits of dairy cows. [file AGE-51-141-s002.doc]

**Table S1 Descriptive statistic of the 29 conformation traits used in the GWAS**

| **Body Confirmation Trait** | **Sample Number** | **Mean**  **Value** | **Minimum Value** | **Maximum Value** | **Standard Derivation** | **Variance** |
| --- | --- | --- | --- | --- | --- | --- |
| Body height, BH | 421 | 7.38 | 2 | 9 | 1.10 | 1.21 |
| Height at front end, HF | 421 | 5.79 | 2 | 8 | 0.86 | 0.74 |
| Body depth, BD | 421 | 6.97 | 2 | 9 | 0.92 | 0.85 |
| Stature, ST | 421 | 7.55 | 5 | 9 | 0.88 | 0.77 |
| Chest width, CW | 421 | 7.29 | 4 | 9 | 1.07 | 1.15 |
| Angularity, AG | 421 | 5.09 | 1 | 8 | 2.13 | 4.53 |
| Loin strength, LS | 421 | 5.55 | 2 | 8 | 1.27 | 1.60 |
| Rump angle, RA | 421 | 5.29 | 1 | 9 | 1.67 | 2.80 |
| Pin width, PW | 421 | 6.69 | 5 | 9 | 0.86 | 0.75 |
| Udder depth, UD | 421 | 3.78 | 1 | 9 | 1.69 | 2.85 |
| Udder texture, UT | 421 | 6.11 | 2 | 9 | 1.47 | 2.15 |
| Median suspensory, MS | 421 | 5.30 | 1 | 8 | 1.79 | 3.20 |
| Fore udder attachment, FUA | 421 | 5.71 | 1 | 9 | 1.81 | 3.29 |
| Front teat placement, FTP | 421 | 4.92 | 1 | 8 | 0.75 | 0.56 |
| Attachment height, AH | 421 | 6.14 | 1 | 9 | 1.23 | 1.51 |
| Attachment width, AW | 421 | 5.94 | 1 | 9 | 1.57 | 2.45 |
| Rear teat placement, RTP | 421 | 5.81 | 1 | 9 | 1.22 | 1.49 |
| Teat length, TL | 421 | 3.95 | 1 | 7 | 0.84 | 0.70 |
| Foot angle, FA | 421 | 4.89 | 1 | 8 | 1.18 | 1.39 |
| Heel depth, HD | 421 | 5.54 | 1 | 8 | 1.24 | 1.55 |
| Bone quality, BQ | 421 | 6.11 | 2 | 9 | 1.28 | 1.65 |
| Rear legs-side view, RLSV | 421 | 4.05 | 1 | 8 | 1.29 | 1.67 |
| Rear legs-rear view, RLRV | 421 | 5.28 | 1 | 9 | 1.73 | 2.99 |
| Stature score, SS | 421 | 89.49 | 78.80 | 95.60 | 2.65 | 7.03 |
| Rump system score, RSS | 421 | 82.00 | 68.40 | 94.00 | 3.63 | 13.21 |
| Mammary system score, MSS | 421 | 79.97 | 64.20 | 91.50 | 6.04 | 36.52 |
| Udder system score, USS | 421 | 78.98 | 58.10 | 89.30 | 4.83 | 23.35 |
| Feet and legs score, FLS | 421 | 82.04 | 68.40 | 94.00 | 3.36 | 11.30 |
| Final score, FS | 421 | 81.66 | 71.60 | 88.20 | 2.73 | 7.47 |

**Table S2 Distribution of SNP markers by chromosomes before and after quality control**

| **Chromosome** | **Physical Length**  **(Mb)** | **Before Quality Control** | |  | **After Quality Control** | |
| --- | --- | --- | --- | --- | --- | --- |
|  |  | **SNPs Number** | **Density(SNP/Kb)** |  | **SNPs Number** | **Density(SNP/Kb)** |
| 1 | 158.3 | 1379 | 8.7 |  | 1182 | 7.3 |
| 2 | 137.1 | 1155 | 8.4 |  | 899 | 6.4 |
| 3 | 121.4 | 1097 | 9 |  | 880 | 7.2 |
| 4 | 120.8 | 1113 | 9.2 |  | 845 | 7.1 |
| 5 | 121.2 | 1330 | 11 |  | 1059 | 8.7 |
| 6 | 119.5 | 1175 | 9.8 |  | 962 | 8.1 |
| 7 | 112.6 | 1035 | 9.2 |  | 819 | 7.3 |
| 8 | 113.4 | 990 | 8.7 |  | 707 | 6.2 |
| 9 | 105.7 | 963 | 9.1 |  | 739 | 6.9 |
| 10 | 104.3 | 946 | 9.1 |  | 767 | 7.2 |
| 11 | 107.3 | 1027 | 9.6 |  | 834 | 7.8 |
| 12 | 91.2 | 852 | 9.3 |  | 656 | 7.2 |
| 13 | 84.2 | 846 | 10 |  | 579 | 6.9 |
| 14 | 84.6 | 886 | 10.5 |  | 668 | 7.9 |
| 15 | 85.3 | 837 | 9.8 |  | 699 | 8.2 |
| 16 | 81.7 | 759 | 9.3 |  | 596 | 7.3 |
| 17 | 75.2 | 734 | 9.8 |  | 602 | 8 |
| 18 | 66 | 791 | 12 |  | 710 | 10.5 |
| 19 | 64.1 | 764 | 11.9 |  | 665 | 10.4 |
| 20 | 72 | 743 | 10.3 |  | 667 | 9.1 |
| 21 | 71.6 | 731 | 10.2 |  | 585 | 8.2 |
| 22 | 61.4 | 599 | 9.8 |  | 528 | 8.4 |
| 23 | 52.5 | 595 | 11.3 |  | 523 | 10 |
| 24 | 62.7 | 625 | 10 |  | 494 | 7.9 |
| 25 | 42.9 | 517 | 12.1 |  | 468 | 10.7 |
| 26 | 51.7 | 531 | 10.3 |  | 464 | 8.9 |
| 27 | 45.4 | 458 | 10.1 |  | 393 | 8.6 |
| 28 | 46.3 | 505 | 10.9 |  | 443 | 9.5 |
| 29 | 51.5 | 589 | 11.4 |  | 518 | 9.9 |
| X | 148.8 | 1404 | 9.4 |  | 681 | 4.5 |

**Table S3. The chromosomal distribution of significant SNPs that associated with body confirmation traits**

| **Chromosome** | **No. of significant SNPs** | **Associated traits^1^ (SNP No.)** |
| --- | --- | --- |
| 1 | 4 | RA(4) |
| 2 | 1 | RA (1) |
| 3 | 0 | - |
| 4 | 1 | RA (1) |
| 5 | 2 | LS (1); MS (1) |
| 6 | 4 | FS (1); RA (2); USS (1) |
| 7 | 4 | LS (1); RA (3) |
| 8 | 1 | RA (1) |
| 9 | 4 | AW (1); MS (1); RA (1); RTP (1) |
| 10 | 5 | ST (1); RA (3); STS (1) |
| 11 | 3 | FS (1); BH (2) |
| 12 | 0 | - |
| 13 | 3 | FS (1); MSS (1); BH (1) |
| 14 | 0 | - |
| 15 | 1 | MSS (1) |
| 16 | 3 | FS (3) |
| 17 | 3 | CW (1); MS (1); STS (1) |
| 18 | 7 | AW (1); FS (2); MSS (1); RA (3) |
| 19 | 3 | AW (1); ST (1); FS (1) |
| 20 | 0 | - |
| 21 | 6 | AW (5); FS (1) |
| 22 | 2 | RA (2) |
| 23 | 1 | BH (1) |
| 24 | 1 | RA (1) |
| 25 | 6 | FS (2); RA (2); UD (2) |
| 26 | 3 | AW (1); FS (1); RA (1) |
| 27 | 1 | BH (1) |
| 28 | 1 | LS (1) |
| 29 | 1 | RA (1) |
| 30 ^2^ | 8 | AG (3); FS (1); MS (1); MSS (1); RA (2) |

^1^ AG, angularity; AW, attachment width; BH, body height; CW, chest width; FS, final score; LS, loin strength; MS, median suspensory; MSS, mammary system score; RA, Rump angle; RTP, rear teat placement; ST, stature; STS, stature score; UD, udder depth; USS, Udder system score.

^2^ Chromosome 30 represents the X sexual chromosome.

**Table S4. The SNPs identified associated with multiple body confirmation traits of dairy cows.**

| **SNP name** | **Chromosome** | **Position** | **Gene** | **Distance** | **MAF** | **Associated traits^1^** | ***P*_value** |
| --- | --- | --- | --- | --- | --- | --- | --- |
| BovineHD0600000461 | 6 | 1770665 | *-* | Within | 0.3900 | Final score (FS) | 1.75E-06 |
|  |  |  |  |  |  | Udder system score (USS) | 1.03E-07 |
| BovineHD1900007686 | 19 | 26049717 | *AIPL1* | D: 7711 | 0.03628 | Stature (ST) | 2.15E-06 |
|  |  |  |  |  |  | Final score (FS) | 7.31E-07 |
| ARS-BFGL-NGS-41612 | 11 | 80730546 | *KCNS3* | U: 112015 | 0.1485 | Final score (FS) | 4.61E-07 |
|  |  |  |  |  |  | Body height (BH) | 4.93E-07 |
| BovineHD1700010514 | 17 | 38504014 | *LOC512119* | D: 176780 | 0.09545 | Chest width (CW) | 1.34E-07 |
|  |  |  |  |  |  | Stature score (STS) | 2.42E-08 |
| BovineHD3000037672 | X | 132705219 | *LOC786725* | U: 54284 | 0.4490 | Angularity (AG) | 5.10E-08 |
|  |  |  |  |  |  | Mammary system score (MSS) | 7.03E-08 |
| ARS-BFGL-NGS-109467 | 13 | 28331553 | *SEPHS1* | D: 16954 | 0.4388 | Final score (FS) | 1.80E-06 |
|  |  |  |  |  |  | Mammary system score (MSS) | 5.49E-07 |

^1^ The distance from the SNP locus to the gene (unit: bp); D and U indicate that the SNP site are located in the downstream and upstream of the gene, respectively; Within indicates that the SNP locus is located within the gene.

^2^ MAF means Minor Allele Frequency of the SNP.

**Table S5. Multiple SNPs locates in the *SLC24A4* gene region were significantly associated with body confirmation traits of dairy cows.**

| **SNP name** | **Position** | **Chromosome** | **Distance from *SLC24A4* gene ^1^** | **MAF ^2^** | **Associated traits** | ***P*_value** |
| --- | --- | --- | --- | --- | --- | --- |
| BovineHD2100016535 | 57552028 | 21 | U: 44433 | 0.234 | Final score (FS) | 8.46E-07 |
| BovineHD2100016545 | 57583470 | 21 | U: 12991 | 0.4478 | attachment width (AW) | 2.26E-06 |
| BovineHD2100016546 | 57584406 | 21 | U: 12055 | 0.4477 | attachment width (AW) | 2.33E-06 |
| BovineHD2100016549 | 57587712 | 21 | U: 8749 | 0.4478 | attachment width (AW) | 2.26E-06 |
| BovineHD2100016561 | 57620878 | 21 | Within | 0.2823 | attachment width (AW) | 9.13E-07 |
| BovineHD2100016563 | 57623572 | 21 | Within | 0.2823 | attachment width (AW) | 9.13E-07 |

^1^ The distance from the SNP locus to the gene (unit: bp); U indicates that the SNP site is located in the upstream of the gene; Within indicates that the SNP locus is located within the gene.

^2^ MAF means Minor Allele Frequency of the SNP.
